# Does the Podcast Video Playback Speed Affect Comprehension for Novel Curriculum Delivery? A Randomized Trial

**DOI:** 10.5811/westjem.2017.10.36027

**Published:** 2017-12-05

**Authors:** Kristine Song, Amit Chakraborty, Matthew Dawson, Adam Dugan, Brian Adkins, Christopher Doty

**Affiliations:** * University of Kentucky, Department of Emergency Medicine, Lexington, Kentucky; † Stanford University, Department of Radiology, Palo Alto, California

## Abstract

**Introduction:**

Medical education is a rapidly evolving field that has been using new technology to improve how medical students learn. One of the recent implementations in medical education is the recording of lectures for the purpose of playback at various speeds. Though previous studies done via surveys have shown a subjective increase in the rate of knowledge acquisition when learning from sped-up lectures, no quantitative studies have measured information retention. The purpose of this study was to compare mean test scores on written assessments to objectively determine if watching a video of a recorded lecture at 1.5× speed was significantly different than 1.0× speed for the immediate retention of novel material.

**Methods:**

Fifty-four University of Kentucky medical students volunteered to participate in this study. The subjects were divided into two separate groups: Group A and Group B. Each group watched two separate videos, the first at 1.5× speed and the second at 1.0× speed, then completed assessments following each. The topics of the two videos were ultrasonography artifacts and transducers. Group A watched the artifacts video first at 1.5× speed followed by the transducers video at 1.0× speed. Group B watched the transducers video first at 1.5× speed followed by the artifacts video at 1.0× speed. The percentage correct on the written assessment were calculated for each subject at each video speed. The mean and standard deviation were also calculated using a t-test to determine if there was a significant difference in assessment scores between 1.5× and 1.0× speeds.

**Results:**

There was a significant (p=0.0188) detriment in performance on the artifacts quiz at 1.5× speed (mean 61.4; 95% confidence interval [CI]-53.9, 68.9) compared to the control group at normal speed (mean 72.7; 95% CI−66.8, 78.6). On the transducers assessment, there was not a significant (p=0.1365) difference in performance in the 1.5× speed group (mean 66.9; CI− 59.8, 74.0) compared to the control group (mean 73.8; CI− 67.7, 79.8).

**Conclusion:**

These findings suggest that, unlike previously published studies that showed subjective improvement in performance with sped-up video-recorded lectures compared to normal speed, objective performance may be worse.

## INTRODUCTION

Medical education is a rapidly evolving field that has been using new forms of media and technology to enhance the learning of medical students across the U.S. One of the more prevalent and extensively used advancements is the use of video recording systems. In previous studies, medical students have reported greater subjective benefit from video-recorded lectures than from live lectures.^1^,^2^,^3^ Some of the subjective benefits that students reported included faster knowledge acquisition, better retention of material, more focus, and easier access to additional information.^1^

The advantages to using video-recorded lectures include the ability to rewind, pause and return to finish a lecture later, and watch lectures at faster speeds. In a medical school setting where knowledge of minutiae and comprehension of concepts is paramount to success, the added flexibility that video-recorded lectures provide could be extremely important. As medical education demands countless hours of studying, the biggest advantage may be the ability to watch lectures at faster speeds. As an example, for every hour of material in the traditional classroom setting played at 1.5× speed, a student could save 20 minutes. Therefore, in a typical four-hour morning lecture scenario, a student could save 80 minutes by watching the video at 1.5× speed.

Though the benefits may seem numerous and prior survey studies have shown subjective benefits with lectures played at faster speeds, no quantitative studies to date have measured information retention. The objective of this pilot study was to determine if watching video-recorded lectures at faster speeds compared to the original recording had any effect on the immediate retention of novel learning material.

## METHODS

This was a prospective, single-center, randomized controlled trial, pilot study that presented a novel curriculum to medical students and tested information retention with a short examination. Material presentation and assessments were all done in a single day. The study was approved by the university institutional review board (IRB), as well as by the administration of the medical school involved.

### Video selection and assessment creation

The two new educational subjects chosen were transducers and artifacts. The presented videos were recorded by a nationally recognized emergency ultrasound educator. These two specific videos were chosen because these topics are not covered in the medical school curriculum and, therefore, we believed that the material presented was novel. Subjects were also asked if they had exposure to the material; if they had, they were excluded from the study. Additionally, these videos were similar in length (transducers, 12 minutes; artifacts, 15 minutes) and “factoid heavy,” or in other words, had a wealth of material that could be used to assess student learning.

**Population Health Research Capsule**

What do we already know about this issue?

Previous survey studies have shown a subjective increase in the rate of knowledge acquisition from sped-up lectures. However, no quantitative studies have measured objective retention.

What was the research question?

Would students’ mean test scores differ significantly after watching video lectures at 1.5× speed compared to 1.0× speed?

What was the major finding of the study?

We found that watching a lecture at a faster speed may have a detrimental or no significant effect on learning novel material.

How does this improve population health?

This data is important to modern learners as it challenges the assumption that faster podcast speeds lead to potential time savings for learners.

We created a 23-question multiple-choice assessment for the transducers video, and a 20-question assessment for the artifacts video. Given the pilot nature of the study, the assessments were tested by the two medical students involved in the study as well as by the video creator and content experts to ensure that the two tests were of comparable difficulty levels. To avoid different interpretations of a correct answer, the tests asked about definable facts explicitly stated in the video. The investigators created an answer key for both assessments, which were subsequently reviewed and proofread before submission to the IRB.

### Subject recruitment and privacy

We conducted the experiment in August, at the beginning of the school year, when first-year (M1), second-year (M2) and third-year (M3) medical students had limited exposures to emergency medicine (EM). Subject recruitment was conducted by first sending an email to all four medical school classes. This was then followed by live announcements to each class. All students were told that results on the assessments would have no effect on medical school evaluations or grades and that participation was voluntary. Students who participated were given a $5 Starbucks gift card as a token of appreciation.

Inclusion criteria for subjects included being a medical student and being over the age of 18. The primary exclusion criterion was having been exposed to the presented material before, since prior knowledge of the subjects could skew the results. Additionally, medical students with prior ultrasound experience and students rotating in EM were excluded from the study.

To enforce the exclusion criteria, each participant was asked to enroll via Google Docs and sign an informed consent prior to the study. Each student was asked to answer “yes” or “no” to having previously seen either the transducers video or the artifacts video. Those who answered “yes” received the $5 Starbucks gift card and the opportunity to sit in on the study, but they were excluded from examination and data analysis. To protect the privacy of student performance, each student was identified by his/her student ID number. Demographic data including ethnicity, gender, and year in medical school were also collected. Assessment performance was not shared with the medical school.

### Presentation and assessment

Of the 81 students who signed up for the study, 63 showed up on the day of the experiment. Two were excluded from the examination for having prior knowledge of the material, and seven were excluded from assessment for arriving late. As a result, 54 medical students were included in the final data analysis. Participants were randomized into group A or B by converting the Google docs sign-up document to a Microsoft Excel spreadsheet, and then using the “randomize” function to assign participants into either group A or B. On the date of the experiment, participants could see their group assignment at the check-in desk.

Group A watched the artifacts video at 1.5× speed first and then took the artifacts assessment immediately after the video. Following this, Group A watched the transducers video at normal speed and immediately took the transducers assessment. Group A served as the experimental group for the artifacts video and the control group for the transducers video. Group B watched the transducers video at 1.5× speed first and took the corresponding assessment immediately following the video. Group B then watched the artifacts video at normal speed and took the assessment. Group B served as the experimental group for the transducers video and the control group for the artifacts video.

### Scoring and statistical analysis

Since the two assessments had an unequal number of questions, the scores were converted into a percent correct score. Then the means and standard deviations (SD) of the variable of interest and percentage score were calculated for each assessments (Group A Artifacts, Group A Transducers, Group B Transducers, and Group B Artifacts). Group A Artifacts at 1.5× speed was compared to Group B Artifacts at normal speed using a t-test. Similarly, Group B Transducers at 1.5× speed was compared to Group A Transducers at normal speed using a t-test. To compare the demographic information, such as year in medical school, gender, and ethnicity, the Fisher’s exact test was used. The p-values were used to determine if there was a significant difference in performance for both videos. Statistical significance was set at p < 0.05. All analyses were completed in R version 3.4.1 (R Core Team; Vienna, Austria).

## RESULTS

### Subject data

A total of 54 students participated in the study. Of these, 21 were M1s, 27 were M2s, two were M3s, and four were M4s. Thirty participants were female and 24 were male. Finally, seven participants self-identified as Asian American, one as African American, 45 as Caucasian, and one participant chose not to provide ethnicity (Table 1).

There was a similar distribution of M1, M2, M3, and M4 in each group. Among the 21 M1s, 11 were in Group A and 10 in Group B. Among the 27 M2s, 14 were in Group B and 13 in Group B. Each Group A and B had three M3 and M4 participants.

### Assessment results

The average performance of Group A on the assessment for Artifacts at 1.5× speed ± SD was 61.4 ± 19.3% (95% CI [−53.9, 68.9]). On the Transducers assessment at normal speed, Group A’s mean performance was 73.8 ± 15.6% (95% CI [−67.7, 79.8]) (Table 2).

The average performance of Group B on the assessment for transducers at 1.5× speed was 66.9 ± 17.6% (95% CI [−59.8, 74.0]). For artifacts at normal speed, Group B averaged 72.7 ± 14.6% (95% CI [−66.8, 78.6]).

For both videos, the performance of the control and experimental groups were compared using the t-test, and estimated the effect sizes using Cohen’s d. For the artifacts video, there was a significant difference between the performance at 1.5× speed compared to 1.0× speed (p= 0.0188), along with a moderate effect size (Cohen’s d = 0.654). For the transducers video, a statistically non-significant (p= 0.1365) difference was found in the performance between the two groups along with a small effect size (Cohen’s d = 0.414).

We compared the performance of the control groups of artifacts and transducers videos using the t-test and found no significant difference (p= 0.7965), suggesting that the tests did not differ in difficulty.

## DISCUSSION

We chose to conduct a quantitative analysis of students’ information retention after viewing a sped-up video compared to retention at normal speed because there was a lack of literature regarding the topic. To assess retention, novel education material was presented to the test subjects one at normal speed and another at 1.5× speed and assessed comprehension after each video. Participants overall performed worse on assessments after learning from 1.5× speed compared to 1.0× speed. For the artifacts video, the average test score was 72.7 at 1.0× speed compared to 61.4 at 1.5× speed. For the transducers video, the average test score was 73.8 at 1.0× speed compared to 66.9 at 1.5× speed. Our findings were contrary to previous studies that reported subjective, accelerated learning when learning from videos at faster speeds.

The difference in performance on the artifacts quiz at 1.5× speed compared to the control group was significant. Although the difference in performance of the 1.5× group compared to the control group was not significant for the transducers video, the difference was equivalent to a letter-grade difference.

The discrepancy in relative performance between control and experimental groups for each of the videos may be explained by confounding factors. The main confounding error that may have led to the difference in results was that the artifacts video and assessment may have been more inherently complex in nature compared to the transducers video and assessment. Although the video selection was done deliberately to ensure videos were similar in length and in the amount of fact content covered, retrospectively we realized that for a novice learner, a few of the questions on the artifacts video may have been more conceptual compared to the transducers questions.

For example, many students answered correctly to the question asking about the A line on the artifacts video, much in the same way they answered correctly about the linear transducer having a higher frequency. However, with no prior knowledge of how ultrasound works, many students answered incorrectly on posterior acoustic shadowing vs. enhancement. As one participant later remarked, as a new learner she focused all her cognitive energy learning to associate that stones cause sound waves to reflect back, which made her associate stones with the word “enhancement.” However, someone with a basic concept of ultrasound would have easily picked up that because of this reflection of sound waves off the stone, there would be shadowing of the structures lying posterior to it. We theorize that when learning multiple-step processes such as these, playback speed plays a more significant role than when learning a rote memorization fact.

This data is important to modern learners as it challenges the assumption that faster podcast speeds lead to potential time savings for learners. This time savings is only realized if the retention of the material is comparable.

## LIMITATIONS

Among this study’s limitations was the small sample size of 54 students that limited the power of this study. Also, even though students with prior exposure to subject material were excluded from the study, it was impossible to ensure that the entire study population was naïve to the material. Another limitation was the inability to establish that the two novel subject matters presented were equivalent in complexity, as discussed above. Final noteworthy point was our decision to play the 1.5× speed video before the 1.0× speed. This may have affected performance and impacted the study results.

### Future studies

This study was designed to examine the immediate recall of information after watching a video at 1.5× speed vs. at normal speed. However, to emulate the full utility of video-recorded lectures, students must be given the ability to rewind parts of the lecture they did not understand or re-watch a lecture a second time. A potential study design to examine this could involve giving both the experimental and control groups the same allotted time to learn a lecture while using 1.0× speed or 1.5× speed and comparing their performance. Long-term information retention is another variable that should be assessed. This study only tested immediate recall.

## CONCLUSION

Our study suggests that watching a video lecture at a faster speed may have detrimental or no significant effect on learning novel material. Contrary to previous studies showing subjective improvement in performance with sped-up, video-recorded lectures compared to normal speed, our data showed that immediate retention of novel material at 1.5× speed was worse compared to normal speed.

## Figures and Tables

**Table 1:** Demographic information of study subjects (we used Fisher’s exact test to compare the demographic information).

	Overall	Group A	Group B	P-value
	
No. of participants	54	28	26	N/A
Year of medical school, n (%)				
1st	21 (38.9)	11 (39.3)	10 (38.5)	
2nd	27 (50.0)	14 (50.0)	13 (50.0)	
3rd	2 (3.7)	2 (7.1)	0 (0.0)	
4th	4 (7.4)	1 (3.6)	3 (11.5)	
				0.5224
Gender, n (%)				
Female	30 (55.6)	18 (64.3)	12 (46.2)	
Male	24 (44.4)	10 (35.7)	14 (53.8)	
				0.5224
Race, n (%)				
African American	1 (1.9)	0 (0.0)	1 (3.8)	
Asian	7 (13.0)	4 (14.3)	3 (11.5)	
Caucasian	45 (83.2)	23 (82.1)	22 (84.6)	
No Response	1 (1.9)	1 (3.6)	0 (0.0)	
				1.000

**Table 2:** Quiz results: we used a t-test to compare the mean of 1.5× vs. 1.0× speed. For the artifacts video, the difference in average performance after 1.5× speed compared to 1.0× speed was significant. For transducers, the difference in average performance after 1.5× speed compared to 1.0× speed was not significant.

	Overall	1.0× Speed	1.5× Speed	P-value
Artifacts		(Group B)	(Group A)	
No. of Participants	54	26	28	N/A
Mean	66.9	72.7	61.4	0.0188
Standard Deviation	18.0	14.6	19.3	
95% CI		66.8, 78.6	53.9, 68.9	
Median (Quartiles)	65.0 (56.3, 80.0)	75.0 (65.0, 80.0)	60.0 (50.0, 75.0)	
Transducers				
		(Group A)	(Group B)	P-value
No. of Participants	54	28	26	N/A
Mean	70.5	73.8	66.9	0.1365
Standard Deviation	16.8	15.6	17.6	
95% CI		67.7, 79.8	59.8, 74.0	
Median (Quartiles)	69.6 (56.5, 87.0)	73.9 (64.1, 88.0)	69.6 (52.2, 81.5)	

*CI,* confidence interval.
